# Molecular and serological surveillance of canine enteric viruses in stray dogs from Vila do Maio, Cape Verde

**DOI:** 10.1186/1746-6148-10-91

**Published:** 2014-04-23

**Authors:** Pedro Castanheira, Ana Duarte, Solange Gil, Clara Cartaxeiro, Manuel Malta, Sara Vieira, Luis Tavares

**Affiliations:** 1 Centro de Investigação Interdisciplinar em Sanidade Animal (CIISA), Faculdade de Medicina Veterinária, Universidade de Lisboa, Lisboa, Portugal; 2 Veterinários Sem Fronteiras de Portugal, Faculdade de Medicina Veterinária, Universidade de Lisboa, Lisboa, Portugal; 3 Delegação do Ministério do Desenvolvimento Rural, Ilha do Maio, Cape Verde

**Keywords:** Canine coronavirus, Canine distemper virus, Canine parvovirus, Cape verde, Molecular surveillance

## Abstract

**Background:**

Infections caused by canine parvovirus, canine distemper virus and canine coronavirus are an important cause of mortality and morbidity in dogs worldwide. Prior to this study, no information was available concerning the incidence and prevalence of these viruses in Cape Verde archipelago.

**Results:**

To provide information regarding the health status of the canine population in Vila do Maio, Maio Island, Cape Verde, 53 rectal swabs were collected from 53 stray dogs during 2010 and 93 rectal swabs and 88 blood samples were collected from 125 stray dogs in 2011. All rectal swabs (2010 n = 53; 2011 n = 93) were analysed for the presence of canine parvovirus, canine distemper virus and canine coronavirus nucleic acids by quantitative PCR methods. Specific antibodies against canine distemper virus and canine parvovirus were also assessed (2011 n = 88).

From the 2010 sampling, 43.3% (23/53) were positive for canine parvovirus DNA, 11.3% (6/53) for canine distemper virus RNA and 1.9% (1/53) for canine coronavirus RNA. In 2011, the prevalence values for canine parvovirus and canine coronavirus were quite similar to those from the previous year, respectively 44.1% (41/93), and 1.1% (1/93), but canine distemper virus was not detected in any of the samples analysed (0%, 0/93). Antibodies against canine parvovirus were detected in 71.6% (63/88) blood samples and the seroprevalence found for canine distemper virus was 51.1% (45/88).

**Conclusions:**

This study discloses the data obtained in a molecular and serological epidemiological surveillance carried out in urban populations of stray and domestic animals. Virus transmission and spreading occurs easily in large dog populations leading to high mortality rates particularly in unvaccinated susceptible animals. In addition, these animals can act as disease reservoirs for wild animal populations by occasional contact. Identification of susceptible wildlife of Maio Island is of upmost importance to evaluate the risk of pathogen spill over from domestic to wild animals in Cape Verde and to evaluate the associated threat to the wild susceptible species.

## Background

Over the past few years, efforts have been made towards a better understanding of the health status of animal populations, particularly regarding viral infections. Due to their high mutation rate and replication strategies, viruses are responsible for recently recognized emerging diseases, posing a danger not only to domestic and wild animals, but also to humans [1,2].

The high density of domestic and stray animals in urban areas enables viral dissemination and maintenance in these populations. Consequently, these animals can act as reservoirs of diseases, with the possibility of transmission to wildlife populations through occasional contact.

Canine parvovirus (CPV) was first identified in the late 1970s and was responsible for severe hemorrhagic gastroenteritis and myocarditis in dogs [3]. Parvoviruses are extremely stable in the environment and indirect transmission assumes a critical role in spreading and maintenance of the virus in animal populations, especially in wild carnivores, in which contact rates between animals are lower [4]. Shortly after its initial detection, CPV-2 was replaced by two antigenic variants, CPV-2a and CPV-2b and more recently a third variant was described CPV-2c [5,6].

Canine distemper virus (CDV) is the etiological agent of canine distemper, a highly contagious disease, responsible for high mortality rates in dogs worldwide. Sequence analysis of CDV strains originated in different geographical areas from several animal species, showed that the hemagglutinin gene has undergone a genetic drift according to the geographic location [7,8]. Phylogenetic analysis based on this gene revealed the existence of at least nine strains in different geographical areas, namely America-1, America-2, Asia-1, Asia-2, Europe-1/South America 1, European wildlife, Arctic-like, South America 2 and Southern Africa [9,10].

Canine coronavirus (CCoV) causes a mild to moderate enteritis in dogs and its infection is characterized by high morbidity and low mortality. CCoV is transmitted by faecal-oral route and spreads rapidly through a group of susceptible animals [11]. Stressful environments with large concentrations of animals and poor hygienic conditions, often seen in kennels, favour the development of this disease [12]. Although a higher mortality rate is observed in animals with multiple infections with other pathogens such as CPV-2, canine adenovirus type 1 and CDV, CCoV represents *per si* a major infectious agent responsible for several epidemics [13,14].

Virological surveys are conducted throughout the world, allowing the detection and analysis of a large variety of viruses in different animal populations. In Cape Verde archipelago to our knowledge, no similar study had been conducted so far. In order to detect the presence of canine viruses on Maio island, samples collected from stray dogs from Vila do Maio were tested for canine parvovirus (CPV), canine distemper virus (CDV) and canine coronavirus (CCoV), to estimate the viral prevalence in this population and investigate the role of these animals in the maintenance and potential spread of common viral pathogens.

## Results

Records were only available for the specimens sampled in 2011. Of the 125 dogs, all them of undetermined or mixed breeds, 65 were females (52%) and 57 males (46%). For 3 dogs (2%) no data was registered regarding gender.

Diaharreic feaces were described for 4 animals (3%).Only two dogs had been vaccinated, both with Tetradog® vaccine and no information regarding vaccination of the rest of the animals was available (NA).

The percentage of positivity for CPV-DNA was very similar in the 2010 and 2011 sampling; 23/53 (43.3%) and 41/93 (44.1%, respectively). From the 88 sera sampling collected during 2011, 63 (71.6%) tested positive for CPV antibodies, with 10 animals included in the first ELISA Unit (EU) class (100–1000 EU), 29 in the second EU class (1000–10000 EU) and 24 in the third EU class (>10000 EU) (Tables 1 and 2).

**Table 1:** Results of viral nucleic acid investigation in each year (Number of positive samples/total of samples analyzed

	**2010**	**2011**
CPV DNA	23/53 (43,3%)	41/93 (44,1%)
CDV RNA	6/53 (11,3%)	0/93 (0%)
CcoV RNA	1/53 (1,9%)	1/93 (1,1%)

The percentage of positivity in each group is indicated between brackets.

**Table 2:** Results of serology investigation in 2011 (Number of positive samples/total of samples analyzed)

	**2011**						
	**Ab classes**				**Ab categories**		
CPV	100-1000 EU	1000-10000 EU	>10000 EU	CDV	Low titer	Medium titer	High titer
					(IIF 1/20-1/40)	(IIF 1/80-1/160)	(IIF ≥ 1/320)
n	10/88	29/88	24/88	n	43/88	2/88	0/88
Total	63/88 (71,6%)			Total	45/88 (51,1%)		

**Figure 1:**
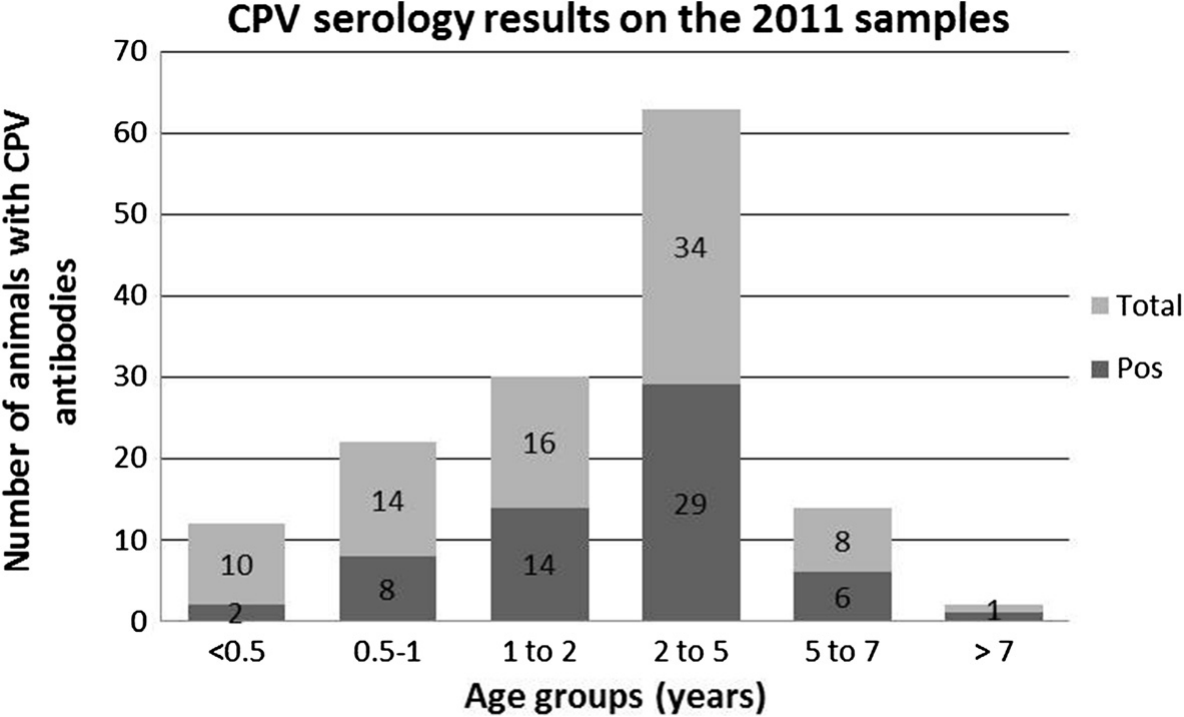
Seropositivity against CPV, accordxing to age class in 2011 sampling.

Antibodies against CPV were detected in 20% of the animals aged less than 6 months (2/10), in 57.1% in dogs aged between 6 months and 1 year (8/14), in 87.5% in dogs with 1 to 2 years (14/16), in 85.3% in dogs with 2 to 5 years (29/34), in 75% in dogs with 5 to 7 years (6/8) and in 1/1 dog older than 7 years (Figure 1). The proportion of seropositive animals was significantly higher in older animals (p < 0.05). No differences were found between the seroprevalence and gender (p > 0.05). From the 56 samples that were tested for virology and serology, 46.6% (26/56) were positive for CPV-DNA and 64.3% (36/56) were seropositive. Out of the 26 dogs that were excreting the virus at the time of collection, 7 were seronegative for CPV specific IgG.

Regarding the 2010 samples tested for CDV-RNA (n = 53), 6 animals were positive (11.3%), of which 2 were also co-infected with CPV. All samples from 2011 were found CDV-RNA negative. As for serology, 45 of the 88 animals sampled during 2011 were seropositive for CDV (51.1%). Two groups were identified according to the antibody (Ab) titer: 1) low Ab titer (IIF values 1/20-1/40: n = 43 (96%)); and 2) medium Ab titer (IIF values 1/80-1/160: n = 2 (4%)).

**Figure 2:**
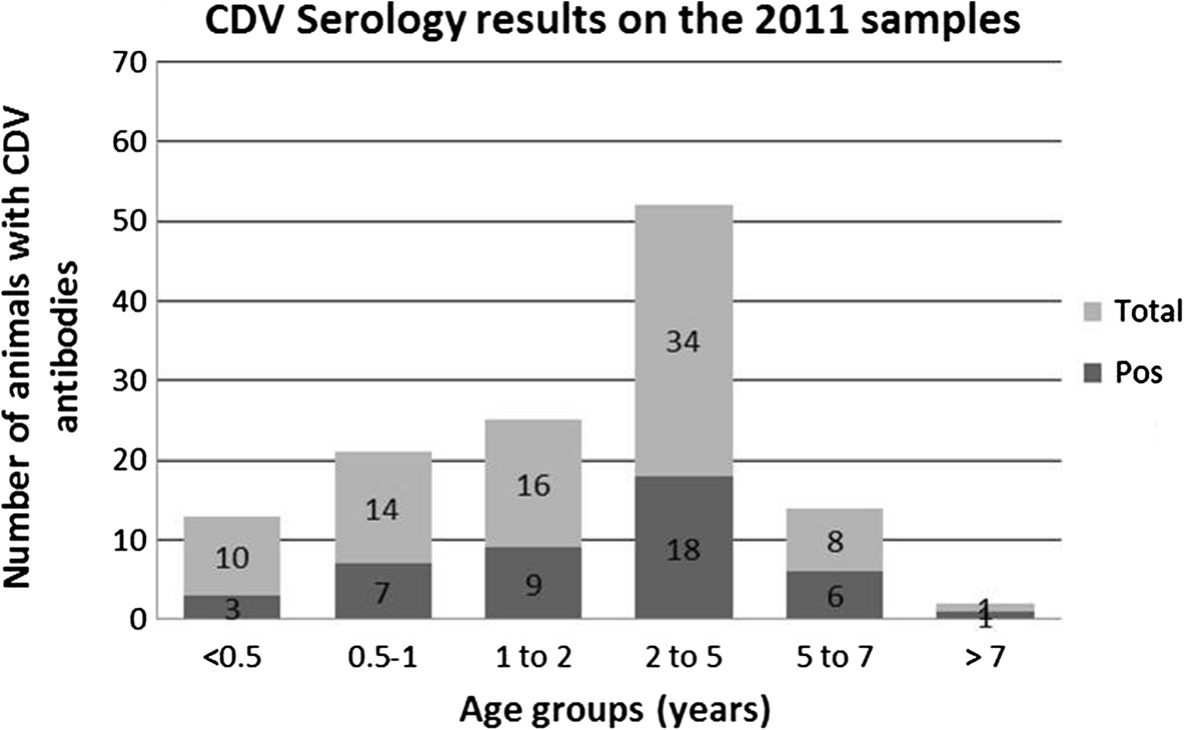
Seropositivity against CDV, according to age class in 2011 sampling.

Antibodies against CDV were detected in 30% of animals aged less than 6 months (3/10), in 50% of dogs aged between 6 months and 1 year (7/14), in 56.3% of dogs with 1 to 2 years (9/16), in 53% of dogs with 2 to 5 years (18/34), in 75% in dogs with 5 to 7 years (6/8) and in 1/1 dog older than 7 years (2011 samples) (Figure 2). Although there was a linear increase of seropositive animals with age, this association was not statistically significant (p > 0.05). The presence of antibodies was independent of gender (p > 0.05).

Only two samples, collected in each year of the survey, tested positive for CCoV-RNA, (2010 (1/53, 1.9%) and 2011(1/93,1.1%)).

## Discussion

This study describes for the first time, the shedding of three common enteric canine viruses, CPV, CDV and CCoV, in 178 stray dogs from Vila do Maio, Cape Verde and reports data on CPV and CDV seroprevalence.

Samples were collected in two consecutive years, 2010 and 2011. Similar frequency of positive CPV animals was found, namely 43.3% in 2010 and 44.1% in 2011, most probably reflecting the high environmental resistance of CPV. Viral transmission and spread of CPV can occur easily, without direct contact between animals. The virus is shed at high titres in faeces and the excretion period may last longer, allowing a higher opportunity for contact between the virus and the new hosts. Although most of the positive dogs did not show signals of diarrhoea, recovered animals may also serve as asymptomatic reservoirs and shed virus periodically, contributing for the persistence and continuous circulation of CPV in the environment, as already reported for cats [15].

Also, the CPV resistance to environmental conditions may explain the high seroprevalence obtained (71.6%), similar to that reported in studies conducted with non-vaccinated dog populations [16,17]. The vast majority of the positive samples presented a high antibody titer (n = 53) (Table 1 and 2), even though it could not be correlated with hemagglutination inhibition values, the gold standard assay for titration of CPV antibodies, however we did not had the possibility to perform this assay. This observation suggests the persistence of the virus in the environment, which is in accordance with the high percentage of carrier animals found (44.1%). Within the 56 animals from which both sample types were obtained, CPV-DNA was detected in 46.6% and seropositivity in 64.3%.

Only 7 of the 26 dogs shedding the virus had no circulating antibodies. It is possible that these animals were at an early stage of infection. Primary IgMs were not investigated by the ELISA kit used in this study, which is specific for IgG detection.

The seroprevalence for CPV was significantly higher in older animals (p < 0.05), most probably reflecting a high likelihood of virus exposure over time. Moreover, as younger dogs are more susceptible to the virus, they can succumb to the disease and therefore be removed from the population. Progressive evolution of the CPV-2 led to the emergence of three antigenic variants, 2a, 2b and 2c, with different properties from the original strain [5]. Monitoring the prevalence of the different CPV in Cape Verde archipelago would be important, not only to assess the distribution of viral variants in this geographic location, but also to understand the evolutionary pattern of the virus in this circunscripted population. In addition, given the high nucleotide substitution rate of CPV, similar to the RNA viruses [18] and the terrain characteristics of Cape Verde as an island its possible transportation through people, goods and infected animals, should be considered.

In 2010 the prevalence of CDV detected by real-time PCR was 11.3% while in 2011 all the samples were CDV-RNA negative. Interestingly, in this year, the seroprevalence obtained for this virus was 51.1%. As these animals were non-vaccinated the explanation for the seropositivity may lie with a previous contact with the virus through ill animals, which could have occurred the previous year.

Several factors may explain the absence of CDV shedding in faeces of dogs sampled in 2011. Taking into account the CDV poor resistance to dry and hot environments, it is possible that the virus did not resist the Cape Verde summer high temperatures, reducing the opportunity for dogs being exposed to the virus [19,20]. In addition, the high mortality rates caused by CDV contribute to the low virus spread in canine populations since the animals that succumbed to infection stop shedding. On the other hand, as the animal density of this city is low compared to larger cities, less contact rates between animals affect the virus maintenance and spread [21]. Still, 51.1% of the animals had anti-CDV antibodies. Comparing the presence of antibodies with the age of the dogs a linear increase in seroprevalence with age was found, which may be related to a possible past CDV outbreak in the population.

Although it is not statistically significant, the higher seroprevalence in older dogs has already been reported [21]. The presence of CDV antibodies was not associated with gender.

All CDV seropositive animals (51.1%) showed a protective antibody titer, according to Twark and Dodds [22], by establishing a comparison between serum neutralization and IIF assays. Although 96% of these animals had low antibody titers (Table 1 and 2), Schultz et al. [23] reported that in actively immunized dogs, either naturally or through vaccination, the antibody titer is not very relevant, provided that is detectable. Nevertheless, and despite the good sensitivity and specificity of ELISA, it would be advisable to test the samples using the serum neutralization test to confirm the presence and titer of neutralizing antibodies, to fully assess the immune status of these animal populations.

Relating to the genetic variability of CDV, it would interesting to identify the different CDV genotypes in positive samples, in order to differentiate between vaccine and field strains, and determine which lineages circulate on this island.

Regarding CCoV, we found a low prevalence of 1.9% in 2010 and 1.1% in 2011, which is in agreement with similar surveys conducted in areas with similar characteristics of Vila do Maio dog population [24,25]. The low prevalence may indicate a low viral circulation, probably due to the virus instability in normal environmental conditions and also to the reduced number viral particles in faeces. As for CPV, dogs recovered from a CCoV infection may function as asymptomatic reservoirs and shed the virus periodically, resulting in a persistent and continued circulation of CCoV in the environment. Available data concerning the epidemiological mechanisms of CCoV suggests that the environment provided by kennels can be fundamental in maintaining this infection in canine populations [25-28].

Seroprevalence against CCoV was not performed due to the rapid decay of antibodies caused by natural exposure [29]. Moreover the available serological assays are based on the identification of antibodies against CCoV-II, and their efficacy is unknown for the detection of CCoV-I antibodies [30], questioning the usefulness of these methods.

## Conclusion

The results presented in this study demonstrate that CPV, CDV and CCoV are circulating in the canine population of Vila do Maio, Cape Verde.

The presence of susceptible animals, the high frequency of infections, the prolonged period of virus shedding and environmental persistence of these agents, especially of CPV, contribute to their continuous circulation in this population. Thus, information regarding the spatial distribution of circulating viruses and the risk factors associated with infections will definitely facilitate the planning of control strategies. To our knowledge no vaccination program is undertaken in this region and its implementation could contribute to increase the immunity of this population, reduce viral circulation, and consequently a decrease the population susceptibility to a future disease outbreaks. Additionally, the absence of control measures may increase the risk of pathogen spill over, either for susceptible newcomers’ hosts or for resident susceptible new hosts as sympatric carnivores’ species.

Due to the wide range of CPV and CDV susceptible hosts, it would be important to identify Maio Island wildlife, to assess the potential risk of infection of these species.

## Methods

### Study population and sampling

Sampling was conducted in Vila do Maio, located on Maio Island, Cape Verde, under a neutering and health surveillance program developed by Veterinarians without Frontiers, Portugal (VSF), in two distinct periods: 2010 with collection of rectal swabs from 53 animals; and 2011, including blood samples (n = 88) and rectal swabs (n = 93) from 125 animals (Table 3). Biological samples were stored under refrigeration until processing.

**Table 3:** Sampling distribution per year of collection

	**Year**	
	**2010**	**2011**
Rectal Swabs (RS)	53	37
Blood	-	32
RS + Blood	-	56
Total	53	125

### Sample processing

Rectal swabs were homogenized in 300 μl of PBS. After centrifugation at 10000xg/10 min, the supernatant was collected and stored at −80°C. Blood samples were centrifuged at 4000xg/10 min to separate plasma from blood cells. Plasma was stored at −20°C until processing.

### PCR assay procedures

For viral nucleic acid extraction, 200 μl of supernatant from each rectal swab, were processed with the Qiamp Minelute kit® (Qiagen, Germany) according to the manufacturer's instructions, for viral DNA and RNA co-extraction. Although using a commercial kit for co- extraction of viral nucleic acids, from a non-recommended biological matrix may imply a reduced nucleic acid yield, it is sufficient in their experience [31,32].

Detection of viral nucleic acids using real-time PCR (qPCR) and real-time rt-PCR (rt qPCR) CPV DNA was amplified by qPCR using TaqMan® Gene Expression 2× Master Mix (Applied Biosystems); CDV and CCoV RNA were amplified by rt-qPCR using the TaqMan® RNA-to-Ct(TM) 1 step kit in a 20 μl reaction with 50 ng of template.

Primers and TaqMan® probes were calculated using the Primer designing tool of NCBI (http://www.ncbi.nlm.nih.gov/tools/primer-blast/). For CPV, primers were based on the nucleotide sequence of the *vp1* gene, available through its access number (AN) AB437433.1. CDV primers were chosen within the nucleocapsid gene (AN JN896987.1) [7] and CCoV primers targeted the highly conserved 7b gene (AN JQ404410.1) as already described by [33] (Table 4). A final concentration of 900 nM for the forward primer, 900 nM of reverse primer and 250 nM of each TaqMan® probe was used (Table 4).

**Table 4:** Nucleotide sequences of the primers and probes used in qPCR (CPV) and rt-qPCR (CDV; CCoV) assays

**Primers/Probe**	**CPV (*vp1 *** **gene AN AB437433.1)**	**CDV (*n *** **gene AN JN896987.1)**	**CCoV (*7b *** **gene (AN JQ404410.1)**
Forward 5’ → 3’	GGGCCTGGGAACAGTCTTGACC (900 nM)	TGGCACTCATTTTGGACATCAA (900 nM)	TGGTCATCGCGCTGTCTACT (900 nM)
Reverse 5’ → 3’	ACCAGAGCGAAGATAAGCAGCG (900 nM)	GCTAACCCAGCTTCCACAATGTA (900 nM)	AGGGTTGCTTGTACCTCCTATTACA (900 nM)
TaqMan® probe 5’ → 3’	FAM CGCCGCTGCAAAAGAACACGACGAAGC TAMRA (250 nM)	FAM TCCCCAGGGAACAAGCCTAGAATTGCT TAMRA (250 nM)	FAM TTGTACAGAATGGTAAGCAC TAMRA (250 nM)
Product	99 bp	100 bp	66 bp

The amplification was performed in the StepOne Plus thermocycler (Applied Biosystems) and the cycling conditions comprised an initial denaturation step at 95°C for 10 minutes, followed by 40 cycles at 95°C for 15 seconds and 1 minute at 60°C. When the template was RNA the amplification cycle included a reverse transcription step at 48°C for 15 minutes.

For CPV tenfold dilutions of CPV-2-780 916 Cornell strain (Tetradog®, Merial) were used as positive control. Regarding CDV, a 287 bp fragment, including the targeted region was amplified from CDV RNA (Caniffa®, Merial) [7] and cloned in pGEM® Teasy vector (Promega) according to the manufactures instructions. The CDV recombinant plasmid was used as positive control. A similar approach was used for CCoV as already described [34].

The assay specificity was confirmed by direct sequencing of the CPV amplicon. For CDV and CCoV sequencing was performed after plasmid cloning. No cross reactivity was detected between CDV/CCoV/CPV. The sensitivity of the rt-qPCR/qPCR for all three agents surpassed the detection of 10 target copies/μl, assessed by conversion of the positive control mass (g/μl) in molecules/μl, based on the following formula: number of copies (molecules/μl) = [mass (g/μl)/(number of base pairs x bp (660)] x Avogadro's number (6,022 x 10^23^).

### Antibody detection

Antibody (Ab) detection was only performed for 2011 serum samples for CPV and CDV using an indirect ELISA kit (Ingezim Canine Parvo 15.CPV.K1® and Ingezim Moquillo 1.5.CDG.K.1®-Ingenasa), for specific IgG detection, according to the manufactures instructions.

This method allowed the quantification of the Ab titer for CPV, using a formula provided by the kit, although it did not specify the correspondence with the hemagglutination inhibition assay for quantification of anti-CPV antibodies. The values were organized in three classes: 1) 100–1000 ELISA Units (EU); 2) 1000–10000 EU and 3) > 10000 EU.

For CDV the OD values had correspondence with the indirect immunofluorescence (IIF) method, and the values were divided into three categories: 1) low titer (IIF values: 1/20-1/40), 2) medium titer (IIF values: 1/80-1/160) and 3) high titer (IIF values: ≥ 1/320).

### Statistical analysis

A possible association between serological findings and the age and gender of the animals was determined by chi-square statistical test (χ^2^) with IBM SPSS Statistics 19.0 software. A P value <0.05 was considered significant.

## Competing interest

The authors declare that they have no competing interests.

## Authors’ contributions

PC, AD and LT were responsible for the conception and the study design and actively participated in the analysis and data interpretation. PC and AD were also involved in the drafting and revision of the article. SG and CC were involved in the data analysis. MM and SV were responsible for the organization of the field work and sample collection.
